# Technologies and techniques to improve precision in breast conserving surgery

**DOI:** 10.1002/jso.27657

**Published:** 2024-08-21

**Authors:** Daniel R. Leff

**Affiliations:** ^1^ Department of Surgery & Cancer Imperial College London London UK; ^2^ Breast Unit Imperial College Healthcare NHS Trust London UK

**Keywords:** breast conserving surgery, confocal laser microscopy, frozen section, imprint cytology, intraoperative margin assessment, MicroCT, margins, mass spectrometry, optical coherence tomography, partial mastectomy, radiofrequency spectroscopy, shave margins

## Abstract

Imprecision in breast conserving surgery results in high rates of take back to theatre for reexcision of margins. This paper reviews the various approaches to improving the precision of oncological margin control in breast conserving surgery. The review describes the rationale for improved tissue characterization over tumor localization and explores technology‐free approaches, as well as progress being made to develop and test innovative technological solutions.

## 1 INTRODUCTION

The current operative platform for breast conserving surgery whereby a surgeon uses electrocautery to remove a breast cancer with a variable rim of healthy breast tissue has arguably barely evolved in last 100 years. Whilst technologies have been developed to better localize the epicenter of breast tumors such a magnetic (e.g., Magseed®, EndoMagnetics) or radiofrequency seeds (e.g., LOCalizer^TM^) to guide occult lesion localization, surgeons still lack the tools to characterize breast tissues in near real‐time or to enable resection routines to be adjusted to optimize oncologic margin control.^1^

**Figure 1:**
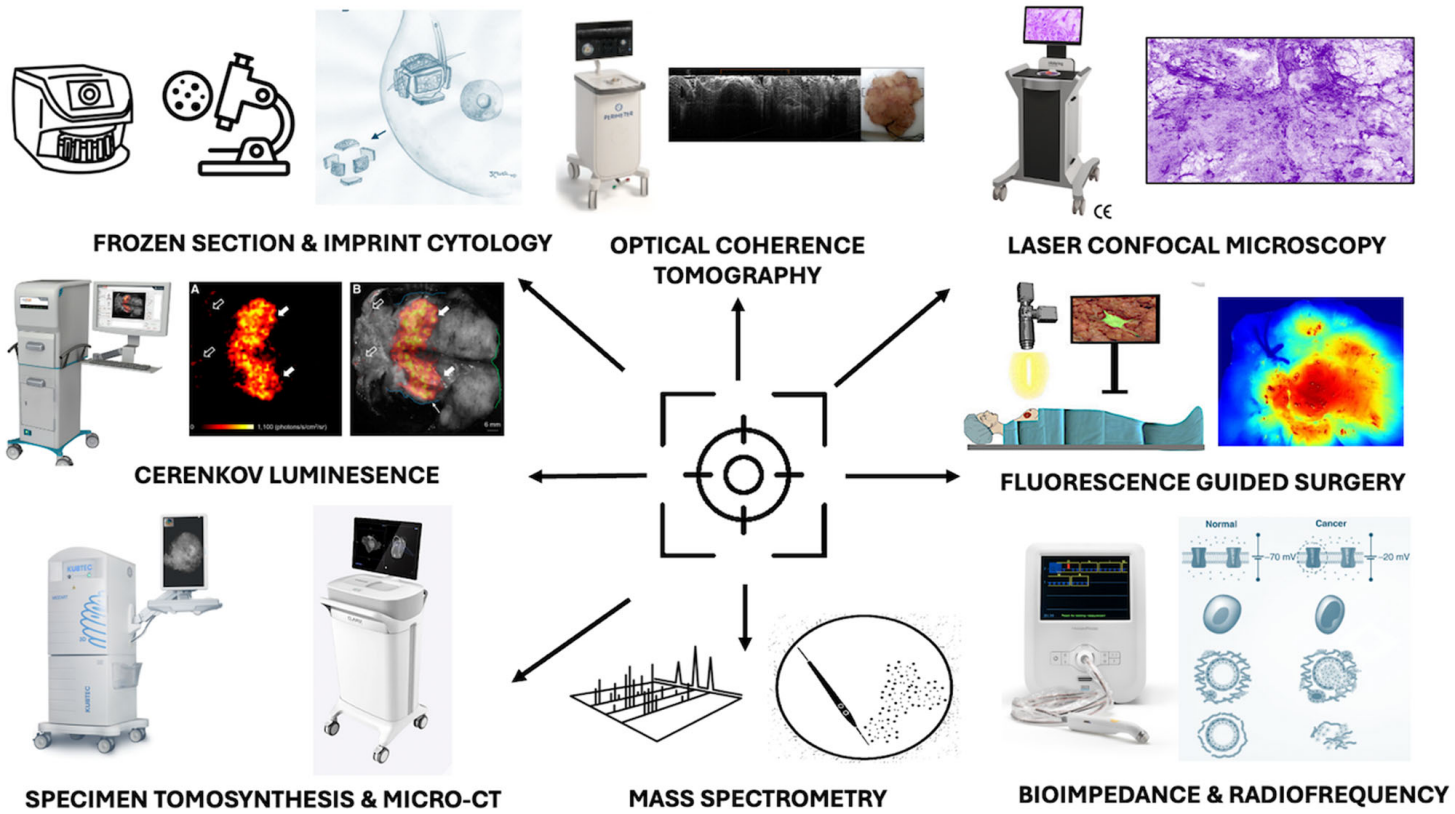
Systems for precision breast conserving surgery. Approaches include immediate cytopathological analysis such as using frozen section or imprint cytology. Improvements in morphological imaging such as with 3D specimen tomosynthesis and microcomputed tomography (Micro‐CT) have been shown to be highly accurate in determining oncological margin status. Functional images such as with position emission tomography (PET) probes as in Cerenkov Luminescence (reproduced with permission from Grootendorst et al.,^10^) or passive or active fluorescence probes (reproduced with permission from Kedrzycki et al.,^11^) may improve detection of macroscopic disease at resection margins. Delivering cellular level resolution to the operating room with optical coherence tomography and confocal microscopy (e.g., HistologTM, Samantree, Lausanne, Switerzland) have been shown to have good accuracy and trials combining these systems with artificial intelligence are likely to lead to rapid insitu diagnostics for improved margin control. Hand‐held probes such as those using radiofrequency technology have been shown in controlled trials to reduce reoperation by 50%. Rapid mass spectral analysis of bovie smoke plume is highly accurate (94% ex‐vivo) and with rapid turnaround time for clinical actionability, but further trials are needed to confirm in‐vivo accuracy and reduction in margin positivity and reoperative interventions.

Current approaches to mapping disease extent and/or to better localizing tumors in‐vivo have failed to provide sufficient levels of precision and as such rates of incomplete excision and reoperative intervention remain unacceptably high.^2^, ^3^ Imprecision is manifest in the human and economic costs of high rates of reoperative intervention for close or positive resection margins.^4^, ^5^, ^6^, ^7^, ^8^ Conversely, imprecision also risks over exuberant resection with excessive normal tissue beyond the recommended margins of clearance resulting in poor cosmesis and breast related quality of life.^9^ Motivated by the need to significantly improve surgical precision there has been a wave of research into new technologies, development of novel systems and early clinical validation towards translation of these innovations (Figure 1). The objective of this review is to provide a narrative summary of the existing literature on technologies for oncological margin control and intraoperative margin assessment, as well as summarize our own efforts towards enhancing the precision of breast cancer surgery.

## 2 WHY PRECISION TECHNOLOGY FOR BREAST SURGERY?

The entire approach from the preoperative work‐up through to the surgical platform for delivering breast conserving surgery must evolve and become more precise. The inability to visualize the breast cancer or detect the cancer–healthy normal interface means the best that any breast cancer surgeon can hope to achieve is to locate the tumor epicenter, estimate the approximate size of the lesion from preoperative imaging and make a best prediction regarding the boundaries of resection. When described as such, it should hardly be surprising that this approach fails to adequately remove the tumor on average 20%–30% of the time.^2^, ^3^, ^12^ In the United States (USA), the American Society of Breast Surgery Mastery Program database suggests an aggregate reoperation rate of 21.6%.^13^ In the United Kingdom (UK), the “getting it right first time” national practice review recently observed a re‐operative intervention rate of 19%,^2^ and others have observed average re‐operative intervention rates of ~30% when ductal carcinoma insitu (DCIS) is present.^3^ Indeed, we recently observed a nine fold odds of margin positivity due to DCIS.^14^ Moreover, the UK national margins audit identified that 3/4 of positive margins for were tumor at the inked margin.^15^

Reoperation interventions have a substantial impact on the patient, the healthcare economy, and providers. Re‐operations on the conserved breast increase the risk of surgical site infection^4^ and lead to greater volumes of tissue being excised resulting in poorer cosmetic outcomes with consequences for quality of life.^6^ Reoperation results in anxiety, distress, delays to receipt of adjuvant therapy and leads to financial toxicity. The economic impact for taxpayers remains unknown, but we estimate median additional costs of £2136/per patient if re‐excision is required.^5^ Based on a crude estimate of the direct costs of reoperation and approximately 11 000 women undergoing re‐excision every 3 years, we estimate triennial costs of £118 m to the UK health service. For the provider, case scheduling is challenging as re‐excision of margin cases need to be accommodated, potentially leading to unavoidable delays to receipt of index surgery in certain patients.

## 3 TRADITIONAL TECHNIQUES

### 3.1 Imaging

Conventional specimen 2D imaging is routine in many Centers in the UK, Europe and USA, and yet has been found to have poor diagnostic accuracy when compared to cytopathological approaches (pooled sensitivity = 0.53, pooled specificity = 0.84 and AUC = 0.73).^16^ In this author's opinion, 2D imaging should only be used to confirm the lesion and/or localization system is in the resected specimen and should not be used to determine whether a resection margin is involved or not.

### 3.2 Immediate cytopathological analysis

Techniques such as frozen section and imprint cytology have high diagnostic accuracy. In systematic review and meta‐analysis, pooled sensitivity was found to be 0.86–0.91 and pooled specificity was 0.95–0.96.^16^ Moreover, several clinical studies highlight the potential of these approaches to be used during the index surgery, to better gauge margin positivity and make appropriate decisions about targeted immediate reexcision of margins.^17^, ^18^, ^19^ Despite these encouraging results, and evidence that these approaches can reduce reoperations,^17^, ^18^, ^19^ they are not widely deployed. Challenges faced by most units is that these techniques are too slow, labour intensive and disrupt the clinical workflow. In the main, Centers do not have the trained personnel to review and report the results of immediate cytopathological techniques in real‐time. None of the so called “big 18” Centres in the London that contribute to national screening external quality assessment use immediate histological analysis.

## 4 TECHNOLOGY FREE APPROACHES

### 4.1 Immediate cavity shave margins

The randomized trials (“SHAVE” & “SHAVE2”) of routine immediate cavity shave margins versus either selective margin shaves or no margin shaves by Chagpar and Colleagues demonstrated that routine cavity shaves attenuate positive margin rates in a range 50%–75%.^20^, ^21^ Routine cavity shaves are a neat, simple and technology free way to reduce reoperative intervention for failed breast conserving surgery. However, arguably this approach is inelegant and, in many ways, epitomizes the imprecision of contemporary breast surgical practice. Evidence suggests that even the rates of positive margins in those undergoing immediate routine shaves is too high (10%–19%).^20^, ^21^ Routine shaves predictably increase the total volume of excision (50% larger in shave group) than in no shave group. Whilst no difference in patient reported outcomes were observed in the SHAVE trial,^20^ the theoretical concern remains that routine cavity shaves may have deleterious effects on cosmesis, aesthetic outcome and breast quality of life. Indeed, in this authors experience this is paramount for simple wide excision and can only be offset through oncoplastic techniques. Such is the imprecision that shave margins risks rendering some patients margin positive on shaves when the original resection margins were adequately clear of disease.

## 5 TECHNOLOGICAL INNOVATIONS

### 5.1 Specimen tomosynthesis

Digital specimen tomosynthesis create 3D images of breast specimens in ~1 mm digital slices. Commercially available systems such as the MOZART® 3D (KUBTEC®) provide surgeons with a far more detailed image of the specimen and target lesion and enables greater interrogation of microcalcifications. Evidence suggests that digital tomosynthesis has improved diagnostic accuracy over standard 2D specimen radiographs (accuracy 2D = 60% vs. 3D = 76%).^22^ This notwithstanding a wide range of sensitivities have been observed in clinical studies.^23^, ^24^, ^25^ In a comparative analysis, Amer et al.,^26^ observed a statistically significant improvement (*p* < 0.0001) in accurately detecting margin positivity with 3D (68% correct) compared to 2D radiographs (44% correct).^26^

### 5.2 Microcomputed tomography (CT)

Micro‐CT are compact scanners that can be placed in the operating room or the grossing area of pathology and provide high‐resolution volumetric images of the resected breast specimen in a nondestructive manner at a resolution in the tens of microns range (70–1000 μm). Commercially available systems include VSI‐360^TM^ (CLARIX Imaging). To date, there have been only a few clinical studies reporting on the diagnostic accuracy of MicroCT, but it seems to compare favorably to specimen mammography.^22^ Compared to conventional 2D radiographs, MicroCT had improved sensitivity (68% vs. 52%) with superior diagnostic accuracy as highlighted by the area under the curve (72% vs. 60%).^22^ It remains to be seen if accurate interpretation of MicroCT images is possibly by surgeons in the operating room and whether the technology can significantly attenuate rates of margin positivity.

### 5.3 Cerenkov luminescence and position emission tomography (PET)

18F‐FDG PET is known to be an accurate tool in the work‐up of breast cancer patients for detection of metastatic disease with high sensitivity (92%–96%) and specificity (84%–91%).^27^ Intra‐operative PET for resection margin control is limited by its relatively poor spatial resolution, and the size and expense of PET cameras. However, PET agents have been found to give off optical photons via a phenomenon known as “Cerenkov Luminsence”.^28^ Optical imaging to detect Cerenkov photons requires highly sensitive cameras synchronized with the linear accelerator (LINAC and Surface Guided Radiation Therapy) to capture and visualize the emitted light. Work conducted by Professor Purusthotham and colleagues initially demonstrated good agreement in margin distance assessed on Cerenkov luminescence imaging (CLI) (κ = 0.81) and established the levels of radiation exposure to surgeons.^10^ In a follow‐up multicenter evaluation, the same group established the diagnostic accuracy of CLI for margin detection showing relatively low sensitivity (46.2%), but good specificity (81.7%) and overall accuracy (80.5%).^29^ Major challenges to be overcome with CLI the optimal site for nanocolloid injection, false positives with nontumor related “hot spots”, and the need for tracers with even greater specificity than [18F]FDG.^29^

### 5.4 Contrast enhanced fluorescence guided surgery

In fluorescence guided surgery, specialized cameras are used in conjunction with fluorescent probes to visualize malignant tissue intraoperatively.^11^ The fluorescence probes accumulates in cancerous tissue either by passively leaking into the tumor microenvironment due to defects in endothelial cells that leads to accumulation into tumor vessel walls (so called the “enhanced permeation and retention” effect), or by actively targeting receptors or enzymes. In a series of studies using the passive agent indocyanine green (ICG) and a custom built “GLOW” camera, our team have shown modest sensitivity (69%–75%) and specificity (89%–97%) depending on the timing of ICG administration and tumor contrast to background tissue metric analysis.^30^, ^31^ Similar diagnostic accuracies have been observed with other passive probes such as with 5‐aminolevulinic acid which fluoresces after conversion to protoporphyrin IX in cancer cells. Ottolino‐Perry et al.,^32^ achieved sensitivity of 65%–68% and specificity of between 80% and 85%.^32^

Our recent review of fluorescence guided breast cancer surgery highlighted that few studies using targeted probes have been able to achieve the minimum diagnostic accuracy for clinical adoption.^11^ In a series of studies exploring utility of LUM015 which is cleaved and activated by cathepsin, Smith et al.,^33^ demonstrated good tumor to background contrast (TBR = 4.22 − 4.70).^33^ In a follow‐up prospective single arm nonrandomized study the same team used the fluorescence agent gegulicainine to guide selective shave margins and found it avoided re‐excision in 19% but missed positive margins in 23.7%.^34^ Other attempts at targeted fluorescence imaging for breast cancer include using bevacizumab bound IRDye800CW.^35^, ^36^, ^37^ Of these, the highest accuracies were reported by Koch et al.,^35^ attaining 98% sensitivity and 79% specificity.^35^ Despite progress in fluorescence imaging for breast conserving surgery, there are too few reports of intraoperative use, and further work is required to confirm whether these systems improve the precision of resection and reduce reoperative interventions for positive margins.^11^

### 5.5 Optical coherence tomography (OCT)

OCT capitalizes on near infrared light to generate detailed, real‐time, multidimensional images of cancer tissue samples.^38^ OCT can differentiate cancer from healthy breast due to the unique light scattering properties of cancer cells caused by large nucleus to cytoplastic ratio, cellular and nuclear density.^39^, ^40^ Whilst the whole specimen is illuminated by OCT and its reflection captured by detectors, its limited by depth of penetration of light to a maximum of 2 mm. However, this depth resolution is arguably within the limits of the current clinical guidance on minimum clear margins, which suggest no tumor of ink for invasive disease^41^ and 2 mm for DCIS.^42^ Clinical studies of OCT suggest sensitivities ranging from 70.8% to 100% and specificities of between 76.9% and 81.8%.^40^, ^43^ More recently, machine learning algorithms applied to OCT images of breast tissue specimens has led to accuracies to beyond 96%.^44^ The results of a pivotal clinical trial led by Professor Alastair Thompson involving the commercial OCT system coupled with its latest generation “ImgAssist” artificial technology made by Perimeter Medical Imaging AI are eagerly awaited.

### 5.6 Probe based microconfocal laser endoscopy

Given the accuracy demonstrated with cytopathological techniques such as frozen section it is logical that systems that offer cellular level resolution straight to the operating room could have translational impact. Confocal laser microscopy (CLM) is an optical imaging technique that enables cellular level imaging without complex and time‐consuming embedding and sectioning of fixed tissues. Our team developed a confocal imaging nomenclature to describe the appearances of common breast pathologies on CLM. We found that breast cancer cells appeared as irregular clusters of hyperfluorescent nuclei, distinct from the polygonal honeycomb structure of normal breast tissues.^45^ In a study involving CLM assessment of 350 images evaluated in a blinded fashion, we observed mean diagnostic accuracy for histopathologists and surgeons was 94% and 92% respectively.^45^ Interobserver agreement was “almost perfect” for histopathologists (κ = 0.82) and “substantial” for surgeons (κ = 0.74).^45^

Towards margin assessment, SamanTree Medical SA, Lausanne, Switzerland) have developed Histolog®, a commercial wide field CLM system. To date, studies have compared surgical decision‐making for targeted immediate shave margins based on intraoperative radiographs (standard) versus images obtained using the CLM scanner.^46^ In a study of 40 patients, Sandor et al.,^46^ found that pathologists could identify a substantial number (~58%) of positive margins from CLM appearances.^46^ In a larger study involving 300 margins assessed by surgeons and pathologists blinded to ground truth H&E results, Togawa et al.,^47^ observed sensitivity of 37.5% and specificity of 78.2%.^47^ We are presently conducted an ongoing analysis of 100 partial mastectomy specimens collected in the prospective, multicenter, pilot “HICOSH” study involving Imperial College and Kings College London (NCT04857229).

### 5.7 Radiofrequency spectroscopy

Conceptually, probes held against the edge of the resected partial mastectomy specimen might provide useful information about which resection surfaces, if any, are involved with disease. One approach involves detecting differences in tissue bioimpedance, which is a measure of opposition of the tissue to an applied electrical field. MarginProbe® (Dilon Technologies) is a commercial system that uses radiofrequency spectroscopy to detect cancer based on difference in membrane potentials, cell to cell connectivity, nuclear morphology, and vascularity. Several randomized trials suggest that MarginProbe® may improve oncological margin control and reduce reoperative intervention by as much as 50%.^48^, ^49^ Interestingly, it is worth noting that these results were not mirrored in a recent RCT of MarginProbe® vs standard care in partial mastectomy patients in the UK, in part thought to be due to wide variation in margin positivity in the probe arm (6.5%–33.3%).^50^ ClearEdge^TM^ (LsBiopath) is a similar probe‐based technology that capitalizes on dielectric properties of tissue. A phase II cohort study showed good diagnostic accuracy,^51^ but there have been no randomized controlled trials with clinical outcomes reported to date. Challenges that transcend all probe‐based systems relate to the potential to repeatedly scan and rescan areas already studied, difficulties in interpreting the results of “reads out” and perhaps most critically the potential for spatial misalignment in relating positive margins on the specimens back to accurate locations on the cavity surface.

### 5.8 Mass spectrometry

Mass spectrometry is an analytical tool for measuring mass to charge ratio (m/z) of molecules in tissue. Our team are exploring the potential of mass spectrometry to identify chemical signatures of breast cancer in near‐real‐time for use during breast conserving surgery. In this approach, mass analysis is done on the bovie smoke plume which is a cloud of ionized particles representing the chemical composition of the tissue recently vaporized.^52^ The technique known as rapid evaporative ionization mass spectrometry (REIMS) has shown to detect breast cancer cells accurately and rapidly. In a study involving 1158 samples, we showed REIMS can detect breast cancer with 93.4% sensitivity and 94.9% specificity in on average 1.8 s.^53^ The theoretical advantage of coupling the tissue dissection tool with a tissue characterization tool, as well as the rapidity of the turnaround time for results with REIMS means it could be used to adjust the resection routine in near real‐time as an “intelligent knife” or “iKnife”. Our recent prospective, multicenter, clinical feasibility study “REI‐EXCISE” [NCT03432429] has highlighted the need for further technology development to improve stability and reliability for clinical grade intraoperative mass analysis as well to improve the accuracy in spatially locating where along the resection margin mass spectral signatures are arising. The need for an improved spatially resolved, navigated iKnife has led to the development of an ultrasound guided NAVI‐iKnife which is soon to be tested in clinical trials.^54^

## 6 CONCLUSION

In conclusion, improving the precision of breast conserving surgery will ensure complete oncologic resection whilst also maximizing breast‐related quality of life by preventing excessive removal of normal breast tissues. Traditional techniques such as cytopathological analysis help to reduce positive margins and reoperations but given the time and human resources involved are unlikely to be a tenable solution. Technology free approaches such as routine shaves may reduce reoperative interventions, but the imprecision of this approach is such that much greater volumes of tissue will be removed and some patient's margins will be rendered positive solely on shaves. Novel systems are on the horizon, and these involve improved morphological (MicroCT) or functional images (fluorescence, CLI or PET), provide near cellular level resolution (e.g., OCT, CLM, etc.) or else electromagnetic or chemical sensing of the tissue (e.g., bioimpedance, radiofrequency, or mass spectrometry). Ultimately, it may be a combination of these systems with their complementary strengths that enhances the precision of breast surgery. What is for certain is that for translation these technologies will have to go beyond diagnostic accuracy and be fast, intuitive and integrate easily into the existing clinical workflow.

## CONFLICT OF INTEREST STATEMENT

Daniel Leff is Co‐PI of the Cancer Research UK funded “REI‐EXCISE” trial investigating the role of mass spectroscopy based intelligent knife for precision breast conserving surgery. He is also a Coinvestigator on the NIHR funded i4i study that led to the development of “GLOW” cameras for fluorescence guided breast surgery.

## SYNOPSIS

Reoperations after failed breast conserving surgery due to close/positive margins remains a major healthcare challenge. Tremendous progress has been made in developing technological solutions for precision in oncological margin control. Future platforms are likely to include more detailed intraoperative interrogation of surgical specimens including 3‐dimensional morphological assessment, optical imaging and chemical sensing.

## Data Availability

No original data enclosed as this is a review paper.
